# ACA-28, an anticancer compound, induces Pap1 nuclear accumulation via ROS-dependent and -independent mechanisms in fission yeast

**DOI:** 10.17912/micropub.biology.000711

**Published:** 2023-08-31

**Authors:** Teruaki Takasaki, Reo Obana, Daiki Fujiwara, Naofumi Tomimoto, Golam Iftakhar Khandakar, Ryosuke Satoh, Reiko Sugiura

**Affiliations:** 1 Faculty of Pharmacy, Kindai University, Higashiosaka, Osaka, Japan

## Abstract

The nucleocytoplasmic transport of proteins is an important mechanism to control cell fate. Pap1 is a fission yeast nucleocytoplasmic shuttling transcription factor of which localization is redox regulated. The nuclear export factor Crm1/exportin negatively regulates Pap1 by exporting it from the nucleus to the cytoplasm. Here, we describe the effect of an anti-cancer compound ACA-28, an improved derivative of 1'-acetoxychavicol acetate (ACA), on the subcellular distribution of Pap1. ACA-28 induced nuclear accumulation of Pap1 more strongly than did ACA. ROS inhibitor N-acetyl-L-cysteine (NAC) partly antagonized the Pap1 nuclear accumulation induced by ACA-28. NAC almost abolished Pap1 nuclear localization upon H _2_ O _2_ , whereas leptomycin B (LMB)-mediated inhibition of Pap1 nuclear export was resistant to NAC. Collectively, ACA-28-mediated apoptosis in cancer cells may involve ROS-dependent and -independent mechanisms.

**Figure 1. ACA-28 stimulates nuclear accumulation of Pap1:**
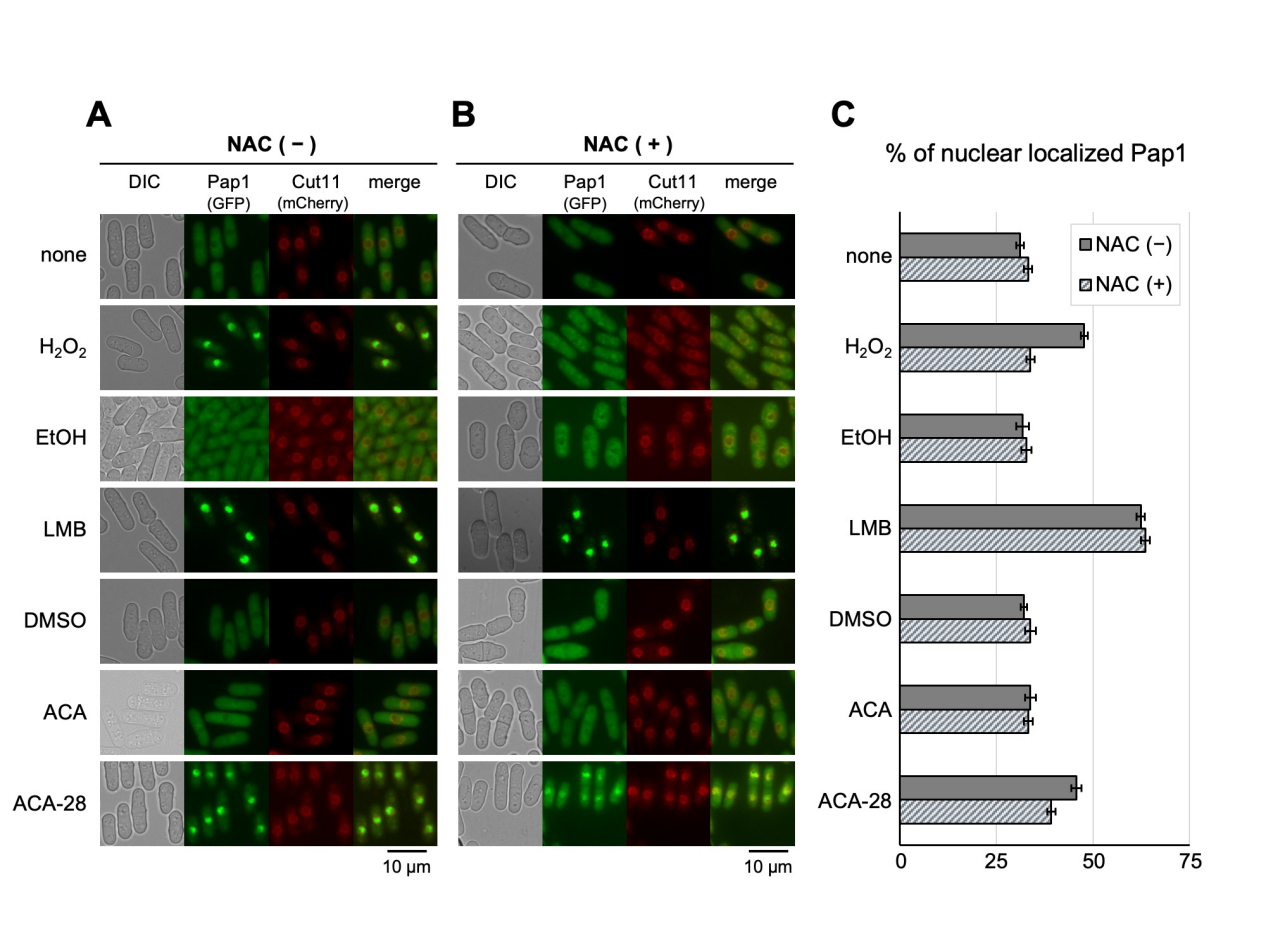
**A:** Representative images of the fission yeast cells co-expressing GFP-tagged Pap1 and mCherry-tagged Cut11 (nuclear envelope marker) under their own promoters treated with each reagent or vehicle at 30˚C for 20 min. Scale bar: 10 µm. **B:** Cells as described in **A** were pretreated with 10 mM of NAC for 60 min at 30˚C followed by incubation with each reagent for 20 min. **C** : Percentages of nuclear-localized Pap1. Each value represents the mean ± 95% confidence interval (CI) of at least three independent experiments in which 25 to 160 cells were analyzed for each specimen.

## Description

The export of proteins between the nucleus and cytoplasm is critically important for normal cell function (Yoneda, 2000) . Deregulation of nuclear-cytoplasmic transport has been detected in diseases, especially cancer (Kau *et al.,* 2004). Thus, nuclear-cytoplasmic transport has attracted strong attention as a therapeutic target for the treatment of cancer. Pap1, a fission yeast bZip transcription factor, is a nucleocytoplasmic shuttling protein and a well-established cargo of Crm1/exportin 1, the nuclear export factor. Pap1 was localized normally in the cytoplasm but was accumulated in the nucleus in response to oxidative stress or when Crm1 was inactivated with leptomycin B (LMB), a specific nuclear export inhibitor (Kudo *et al.,* 1999). Our chemical genetic analysis has previously isolated an anti-cancer compound ACA-28, which has been shown to effectively inhibit the growth of melanoma and pancreatic cancer cells (Khandakar *et al.,* 2022; Satoh *et al.,* 2017). Although ACA-28 has a unique property to preferentially inhibit the proliferation of cancer cells with high ERK activity, how this compound induces apoptosis remains largely unknown. To gain insights into the mechanism of action of ACA-28, here, we use the fission yeast model system by monitoring the effect of ACA-28 on the nuclear-cytoplasmic distribution of Pap1.

To visualize the localization of Pap1, we generated a yeast strain that expresses C-terminally GFP-tagged Pap1 under its promoter at the endogenous genomic locus and crossed with the strain expressing the nuclear envelope marker, Cut11-mCherry (West *et al.,* 1998). Pap1-GFP, which is normally cytoplasmic ( Figure 1A ), was highly accumulated in the nucleus in response to 200 μM H _2_ O _2_ treatment for 20 min ( Figure 1, A and C ). ACA-28 also stimulated Pap1 nuclear accumulation and the percentage of Pap1 localized in the nucleus against that in the whole cell as evaluated by the intensity of GFP fluorescence was significantly increased by the addition of ACA-28 or H _2_ O _2_ as compared with each vehicle ( Figure 1, A and C ). Each vehicle alone (water for H _2_ O _2_ ; DMSO for ACA and ACA-28; EtOH for LMB) did not affect Pap1 distribution ( Figure 1, A and C ). We also analyzed the effect of ACA, the original compound of ACA-28 with inferior apoptosis induction potency against melanoma cells (Satoh *et al.,* 2017), on Pap1 distribution. ACA weakly induced Pap1 nuclear accumulation as compared with the vehicle ( Figure 1, A and C ). LMB strongly stimulated Pap1 nuclear enrichment, consistent with its action to bind to Crm1 thereby preventing the binding of Pap1 to Crm1 ( Figure 1, A and C ) (Toone *et al.,* 1998).

Since Pap1 is activated by various oxidative stresses, we investigated the effect of a ROS inhibitor N-acetylcysteine (NAC) on the subcellular localization of Pap1 treated with ACA-28 and other reagents. NAC significantly reduced the nuclear accumulation of Pap1 induced by ACA-28, although the suppression by NAC was partial ( Figure 1, B and C ). In contrast, NAC almost completely antagonized the nuclear accumulation of Pap1 induced by H _2_ O _2_ . Notably, the LMB-mediated inhibition of Pap1 nuclear export was resistant to NAC ( Figure 1, B and C ). These findings suggest that ACA-28-induced Pap1 nuclear accumulation can be achieved via a ROS-dependent and -independent mechanism. Based on the findings that the original compound ACA showed inferior potency to ACA-28 in terms of cancer cell apoptosis induction and stimulation of Pap1 nuclear accumulation, the superiority of ACA-28 in anti-cancer property may be partly relevant to ROS-dependent mechanism. Intriguingly, the original compound ACA has been reported as an inhibitor for the nuclear export of Rev by binding to the Cysteine-529 residue of CRM1, thereby inhibiting the nuclear export of Rev (Tamura *et al.* 2009). Thus, ACA-28 may also serve as an inhibitor of nuclear export, and this aspect may be reflected by the finding that ACA-28-mediated Pap1 nuclear accumulation showed some resistance to NAC treatment like LMB. Given the considerable number of oncogenes or oncosuppressor genes including p53 are the cargo for the nuclear export receptor CRM1/exportin 1 (Nie *et al.,* 2007), further clarification of the mode of action of ACA-28 might help cancer therapeutics.

## Methods

**Yeast strains and molecular biology**

The *S. pombe* strains used in this study are listed in the Reagents section. C-terminal GFP-tagging of the *pap1 ^+^* gene at the normal chromosomal location was performed by the PCR-based approach described in (Bähler *et al.,* 1998).

**Yeast culture and microscopy**

Yeast strain SP3398 harboring plasmid that contains *LEU2* gene was grown in Edinburgh minimal medium (EMM) in conical tubes at 30°C to mid-log phase and the aliquots of the culture were treated with 200 µM H _2_ O _2_ , 100 ng/ml of leptomycin B (LMB), 32 µM ACA, or 32 µM ACA-28 for 20 min at 30°C. N-acetylcysteine (NAC) was added 60 min prior to the treatment with H _2_ O _2_ , LMB, ACA, or ACA-28. The treated cells were harvested by brief centrifugation as described in (Takasaki *et al.,* 2021). Images were acquired using the BZ-X710 microscope (Keyence Corporation, Osaka, Japan) with a fixed shutter speed, and analyzed by the BZX Hybrid Cell Count Software (Keyence Corporation, Osaka, Japan). The relative GFP fluorescence intensity values in the nuclei against the whole cells were calculated by the following equation: % *Nuclear* = ( *Total Nuclear Intensity* ) / ( *Total Cytoplasmic Intensity* + *Total Nuclear Intensity* ) × 100. Regions of nuclei were determined by the fluorescence of Cut11-mCherry.

## Reagents

**Table:** 

**Strain**	**Genotype**	**Reference**
SP2779	* h ^-^ leu1-32 pap1 ^+^ -GFP::KanMX6 *	This study
SP3020	* h ^+^ leu1-32 cut11 ^+^ -mCherry[natMX6] *	(Hayashi *et al.,* 2018)
SP3398	* h ^+^ leu1-32 pap1 ^+^ -GFP::KanMX6 cut11 ^+^ -mCherry[natMX6] *	This study

## References

[R1] Bähler J, Wu JQ, Longtine MS, Shah NG, McKenzie A 3rd, Steever AB, Wach A, Philippsen P, Pringle JR (1998). Heterologous modules for efficient and versatile PCR-based gene targeting in Schizosaccharomyces pombe.. Yeast.

[R2] Hayashi T, Teruya T, Chaleckis R, Morigasaki S, Yanagida M (2018). S-Adenosylmethionine Synthetase Is Required for Cell Growth, Maintenance of G0 Phase, and Termination of Quiescence in Fission Yeast.. iScience.

[R3] Kau TR, Way JC, Silver PA (2004). Nuclear transport and cancer: from mechanism to intervention.. Nat Rev Cancer.

[R4] Khandakar GI, Satoh R, Takasaki T, Fujitani K, Tanabe G, Sakai K, Nishio K, Sugiura R (2022). ACAGT-007a, an ERK MAPK Signaling Modulator, in Combination with AKT Signaling Inhibition Induces Apoptosis in KRAS Mutant Pancreatic Cancer T3M4 and MIA-Pa-Ca-2 Cells.. Cells.

[R5] Kudo N, Taoka H, Toda T, Yoshida M, Horinouchi S (1999). A novel nuclear export signal sensitive to oxidative stress in the fission yeast transcription factor Pap1.. J Biol Chem.

[R6] Nie L, Sasaki M, Maki CG (2007). Regulation of p53 nuclear export through sequential changes in conformation and ubiquitination.. J Biol Chem.

[R7] Satoh R, Hagihara K, Matsuura K, Manse Y, Kita A, Kunoh T, Masuko T, Moriyama M, Moriyama H, Tanabe G, Muraoka O, Sugiura R (2017). Identification of ACA-28, a 1'-acetoxychavicol acetate analogue compound, as a novel modulator of ERK MAPK signaling, which preferentially kills human melanoma cells.. Genes Cells.

[R8] Takasaki T, Tomimoto N, Ikehata T, Satoh R, Sugiura R (2021). Distinct spatiotemporal distribution of Hsp90 under high-heat and mild-heat stress conditions in fission yeast.. MicroPubl Biol.

[R9] Tamura S, Shiomi A, Kaneko M, Ye Y, Yoshida M, Yoshikawa M, Kimura T, Kobayashi M, Murakami N (2009). New Rev-export inhibitor from Alpinia galanga and structure-activity relationship.. Bioorg Med Chem Lett.

[R10] Toone WM, Kuge S, Samuels M, Morgan BA, Toda T, Jones N (1998). Regulation of the fission yeast transcription factor Pap1 by oxidative stress: requirement for the nuclear export factor Crm1 (Exportin) and the stress-activated MAP kinase Sty1/Spc1.. Genes Dev.

[R11] West RR, Vaisberg EV, Ding R, Nurse P, McIntosh JR (1998). cut11(+): A gene required for cell cycle-dependent spindle pole body anchoring in the nuclear envelope and bipolar spindle formation in Schizosaccharomyces pombe.. Mol Biol Cell.

[R12] Yoneda Y (2000). Nucleocytoplasmic protein traffic and its significance to cell function.. Genes Cells.

